# Techniques and Outcomes of Endoscopic Decompression Using Transanal Drainage Tube Placement for Acute Left-sided Colorectal Obstruction

**DOI:** 10.4021/gr233w

**Published:** 2010-09-20

**Authors:** Yasuyuki Ichise, Akira Horiuchi, Yoshiko Nakayama, Naoki Tanaka

**Affiliations:** a Digestive Disease Center, Showa Inan General Hospital, 3230 Akaho, Komagane, Japan; b Department of Pediatrics, Shinshu University School of Medicine, Shinshu University Graduate School of Medicine, Matsumoto, Japan; c Department of Metabolic Regulation, Shinshu University Graduate School of Medicine, Matsumoto, Japan

**Keywords:** Colonoscopy, Colorectal obstruction, Transanal drainage tube

## Abstract

**Background:**

If it is possible, endoscopic decompression for acute left-sided colorectal obstruction will be effective in critically ill patients. This study was to evaluate the techniques and outcomes of transanal drainage tube placement following urgent colonoscopy in management of acute left-sided colorectal obstruction.

**Methods:**

From January 2000 to December 2009, 69 consecutive patients (36 males, age 38 to 94, mean = 71) were hospitalized because of acute left-sided colorectal obstruction. Urgent colonoscopy was performed within 12 hours of entry for diagnosis and treatment (mean time, 6.5 hours). Endoscopic decompression using a transanal drainage tube was attempted. Clinical success, methods used, and complications were retrospectively evaluated.

**Results:**

The cause of obstruction was colorectal carcinoma in 66 patients (96%). The site of obstruction was sigmoid colon in 37 (54%), rectum in 20 (29%), and descending colon in 12 (17%). Out of 69 patients, endoscopic decompression using the transanal drainage tube was successful in 66 (96%). The use of combination of transanal drainage tube and the equipped guidewire enabled endoscopic decompression was successful in 45 patients (65%), though a small-diameter upper endoscope was used in 2 patients to introduce the guidewire beyond the obstruction. Perforation during the placement developed in 2 patients and one patient was unsuccessful.

**Conclusions:**

Transanal drainage tube placement following urgent colonoscopy was effective in the management of acute left-sided colorectal obstruction. In the majority of patients, the materials and methods used for the transanal drainage tube placement were simple and easy.

## Introduction

Urgent colonoscopy is commonly used for severe lower gastrointestinal bleeding and sigmoid volvulus [1-3]. The diagnosis of acute colorectal obstruction, a life-threatening condition, has been simplified by abdominal computed tomography (CT), which enables acute colorectal obstruction to be discriminated from an obstruction of the small intestine [4, 5]. A precise diagnosis offers gastroenterologists and surgeons the opportunity to potentially manage these conditions endoscopically and prevent emergency surgery [6]. For example, self-expanding metallic stents (SEMS) have been used to relieve malignant colorectal obstruction either as a palliative treatment or a bridge to surgery [7-15]. Endoscopic decompression using a transanal drainage tube (TDT) has also been reported for management of acute colorectal obstruction [16-23]. With regard to a bridge to elective surgery the TDT placement seems to be safer and more cost-effective than SEMS placement [7-23]. Therefore, we have been primarily used the TDT placement as an alternate to emergency surgery for acute left-sided colorectal obstruction.

The aim of the present study was to evaluate the techniques and outcomes of TDT placement following urgent colonoscopy in management of acute left-sided colorectal obstruction.

## Patients and Methods

### Patients

We retrospectively reviewed cases of acute colonic obstruction occurring from January 2000 to December 2009 among patients hospitalized for acute colorectal obstruction at Showa Inan General Hospital. Patients were identified from a colorectal obstruction Database in which all patients underwent surgery and/or colonoscopy. Urgent colonoscopy was defined as colonoscopy performed within 12 hours after diagnosis of acute colorectal obstruction. For all patients who underwent urgent colonoscopy the TDT placement have been attempted. A cleansing enema was only performed when patients were in stable condition. Written informed consent was obtained from each. This retrospective study was approved by the ethics committee at Showa Inan General Hospital.

### TDT placement

The TDT used in the present study was a Dennis^®^ Colorectal Tube, 7.3 mm (22 Fr) in outer diameter and 120 cm in length (Nippon Sherwood, Tokyo, Japan) [18, 19]. A flexible tapered tip is attached to the distal end of the tube. Six holes are present on the side of the tube for decompression. A 0.052-inch guidewire, 350 cm in length, is equipped with the TDT. The TDT is a single use device. The three techniques we used as follows for the management of acute colorectal obstruction have previously been reported [19-21]. A colonoscope (CF-230I, CF-240I, PCF-Q260AI; Olympus, Tokyo, Japan) was inserted and advanced to the site of the tumor. Water-soluble contrast material was injected proximal to the stricture. Carbon dioxide instead of room air was used to alleviate the obstructive symptom due to a complete obstruction.

### Method A

A black hole or small gas bubbles escaping from the obstructed segment identify the obstructed lumen. Under fluoroscopic and endoscopic guidance, the 0.052-inch guidewire was introduced through the tumor beyond the point of obstruction. After the guidewire was positioned, the colonoscope was withdrawn. Under fluoroscopic control, a well-lubricated TDT was introduced over the guidewire and advanced beyond the tumor. The balloon at the tip of the TDT was insufflated with 30 ml of saline and the TDT was fixed. The immediate escape of air and liquid feces through the tube indicated successful decompression. In some technically difficult cases the additional use of a small-diameter upper endoscope (outer diameter 6.5 mm) (GIF-XP260, Olympus, Tokyo, Japan) was useful.

### Method B

When the obstructed lumen could not be detected, a hydrophilic biliary guidewire preloaded through a standard biliary catheter (2.7 mm, 8 Fr) was used to traverse the stricture. Once the wire was passed through the stricture and was recognized fluoroscopically by the anatomically correct position of the wire passing into an air-filled, dilated proximal bowel, the catheter was advanced over the guidewire through the lesion. At this point, the biliary catheter was exchanged for the guidewire catheter in order to use the 0.052-inch guidewire. The TDT was then introduced over the guidewire and advanced beyond the tumor.

### Method C

When it is difficult to insert the TDT beyond the stricture, the use of dilator (26 Fr) (Create Medic Co., Yokohama, Japan) was added to Method B before the insertion of the TDT.

### Outcomes

The main study outcomes were clinical success, the method used and complications. Clinical success was defined as TDT placement with adequate stricture coverage and then relief of colorectal obstructive symptoms maintained without procedure-related complications. Colorectal obstructive symptoms included abdominal pain, bloating, vomiting, and constipation. The incidence of procedure-related complications was evaluated.

### Follow-up

Immediately following the TDT placement, the obstructed colon was irrigated using approximately 10 liters of warm tap water. This typically required at least 1 hour to irrigate the obstructed colon. Two days after tube placement, 500 ml of a polyethylene glycol solution can be given orally for adequate cleansing. After the colorectal obstruction was relieved, a barium study of the proximal colon was performed to rule out the possibility of synchronous carcinoma. In our patients, elective surgery or stenting was then selected based on patients’ clinical condition.

## Results

**Figure 1:**
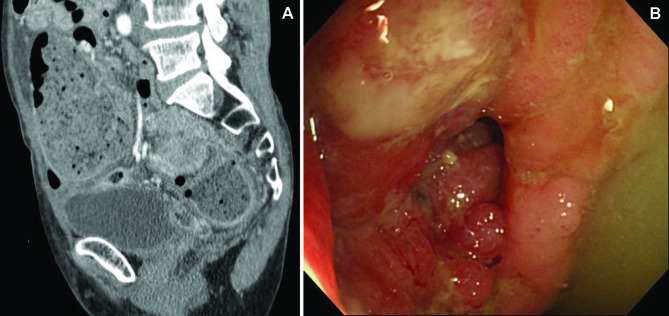
(A) Abdominal CT showing sigmoid carcinoma and the dilated colon; (B) Sigmoid carcinoma showing the obstructed lumen which can be detected.

The study consisted of 69 consecutive patients (36 males and 33 females, age 38 to 94, mean = 71) who admitted at Showa Inan General Hospital for acute left-sided colonic obstruction. All patients had abdominal pain, bloating, and constipation. Physical examination showed a distended and tympanic abdomen. Plain abdominal x-ray revealed a distended large bowel with air-fluid levels. Abdominal CT revealed the site and etiology of acute bowel obstruction. In 69 patients, urgent colonoscopy was performed for diagnosis and treatment within 12 hours of entry (mean time, 6.5 hours) based on CT findings (Fig. 1). Table 1 shows the characteristics of 69 patients enrolled for acute colorectal obstruction. The cause of obstruction was colorectal carcinoma in 66 patients (96%). The site of obstruction was sigmoid colon in 37 (54%), rectum in 20 (29%), and descending colon in 12 (17%).

**Table 1:** Characteristics of 69 Patients Enrolled for Acute Left-sided Colorectal Obstruction

	No. (%)
Age (mean ± SD)*	71 ± 12
Gender (M/F)	36/33 (52/48)
Etiology:	
Colorectal carcinoma	66 (96)
Pancreas carcinoma	1 (1)
Postoperative stenosis	1 (1)
Intussusception	1 (1)
Obstruction location:	
Sigmoid colon	37 (54)
Rectum	20 (29)
Descending colon	12 (17)
Major symptoms:	
Abdominal pain	69 (100)
Constipation	69 (100)
Bloating	69 (100)
Nausea/vomiting	60 (87)

^*^Except for age, the number of the patient is shown.

### Clinical success

**Figure 2:**
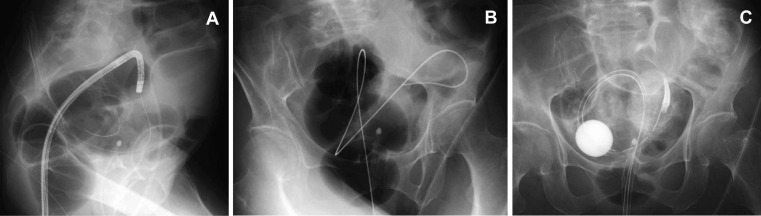
(A) A small-diameter upper endoscope was inserted beyond the obstruction and then the equipped guidewire was introduced; (B) After the endoscope was withdrawn, the guidewire was placed; (C) A transanal drainage tube was placed.

As shown in Table 2, the TDT placement was technically successful in the 66 of 69 patients (96%). The average duration of the tube placement procedure was 27 min. However, the average duration was 42 min for the first 5 cases compared to 17 min of the last 5 cases. In rectum carcinoma it was easy to encounter the lesion and in the majority of them it was simple to place the tube using method A. In spite of an emergency condition, in 65% patients (45/69) the obstructed lumen was identified and the guidewire was introduced through the tumor following by the TDT placement. In 2 patients out of 45, the use of a small-diameter upper endoscope enabled the procedure to be successful (Fig. 2).

**Table 2:** The Methods of Transanal Drainage Tube Placement Used for 69 Patients Undergoing Urgent Colonoscopy

Obstruction Location	Patient Number	Successful Procedure	Procedure Time (min)	Method A GW+TDT	Method B GC+BGW+TDT	Method C GC+BGW+DL+TDT
Rectum	20	20 (100%)	15 ± 6	16	4	0
Sigmoid colon	35	32 (91%)	33 ± 12	18	14	3
Descending colon	12	12 (100%)	27 ± 12	9	1	2
Sigmoid	2	2 (100%)	25	2^*^	0	0
	69	66 (96%)	27 ± 13	45 (65%)	19 (28%)	5 (7%)

Procedure time (min) is shown as mean ± SD.

GW, guidewire; TDT, transanal drainage tube; GC, guide catheter; BGW, biliary guidewire; DL, dilator.

^*^A small-diameter upper endoscope was used.

A complete obstruction with an obscure obstructed lumen was located in the rectum in 4 patients, in the sigmoid colon in 14 patients, and in the descending colon in 1 patient. In those patients a hydrophilic biliary guidewire preloaded through a standard biliary catheter was used to traverse the stricture. The use of the dilator was required for tube placement in 5 patients (Table 2).

All 66 successful patients showed marked improvement in abdominal symptoms shortly after tube placement and repeat abdominal x-ray showed a reduction of the colonic distention. In 61 patients (92%) who underwent elective surgery for colorectal carcinoma, the mean hospital stay was 28 days (range, 19 - 44 days). The mean period until tube placement before surgery was 7 days (range, 4 - 15 days). No anastomotic leakage or postoperative stenosis occurred after operation. The SEMS placement was used as a final palliative treatment in 3 patients (1 pancreatic carcinoma and 2 colorectal carcinomas). The patency of stents lasted a mean of 158 ± 96 days (range, 67 - 344 days). Postoperative stenosis in one patient and retrograde intussusception in one patient were treated by tube replacement.

### Complications

Perforation occurred in two patients (2.9%) with sigmoid colon carcinoma during the TDT placement. Immediately, emergent surgery was performed. In addition, the tube replacement was necessary in one patient because the balloon of the tube broke 4 days after the initial placement.

## Discussion

Although surgical treatment of acute malignant obstruction of the left colon is problematic due to the poor general condition of patients, lack of bowel preparation, and the urgency of the procedure itself, acute colorectal obstruction remains an important indication for emergency surgery. The mortality rate of emergency surgery for large bowel obstruction has been quoted as ranging 15-40% whereas 5% mortality is achieved in elective cases [24, 25]. In these life-threatening situations surgical procedures may be one-staged (tumor resection and primary anastomosis) or two-staged (emergency resection of primary tumor and a colostomy performed and then secondary anastomosis). However, there is still no consensus which procedure should be preferred because of high morbidity and mortality associated with each procedure [24, 25].

Recently, the development of SEMS for colorectal cancer has been associated with technical and clinical success of 97% and 81% respectively with bridging to elective surgery being achieved in 94% of the patients [15]. Two systematic reviews have conducted that SEMS placement is safe and effective as a bridge to surgery and useful as an option to avoid colostomy [10, 11]. Based on these results, SEMS placement as a bridge to surgery has been largely accepted as an alternate to emergency surgery and has resulted in cost saving related to a reduction in length of hospital stay, the number of surgical procedures, and the requirement for intensive care [10]. In addition, the cost of stoma care in the community can be saved including the direct costs of enterostomal supplies and also the indirect costs associated with loss of work and lower health-related quality of life.

However, perforation is a feared complication of SEMS placement; and perianal pain resulting from rectal wall incarceration of the stent, tenesmus from stent placement too proximal to the anus, bleeding, and SEMS migration all have occurred as device-related complications [10-14]. The usefulness of endoscopic decompression using a TDT has also been reported [18-23]. Tube placement has been complicated by perforation in 0-7% while other complications such as bleeding and migration have not been reported. Tube placement with the TDT may be safer than stent placement. Furthermore, tube placement is much cheaper than stent placement for preoperative treatment as the cost of TDT ($500) is one-fourth of a SEMS ($2000) [18]. In addition, tube placement appears to be easier than the SEMS placement, although both SEMS and TDT have to be placed by a skilled endoscopist [21].

We describe urgent colonoscopy that may help to overcome the emergency situation of acute malignant colorectal obstruction. In about 65% of patients, it was possible to perform the TDT placement with only the materials supplied in the kit (Table 2). The procedure time was within 20 min. These data support the usefulness of endoscopic decompression using the TDT placement for acute left-sided colonic obstruction.

Based on the results of the average procedure times of early and late 5 cases there was a learning curve with the tube placement as well as stenting. In our experiences the technique was gradually improving and the procedure time seemed to become shorter. It is also possible that surgical procedures following stenting may be more difficult than surgery without the stent although this hypothesis remains to be tested prospectively.

The length of mean hospital stay in Japan is typically longer compared to American studies related to difference of the insurance system between Japan and the US. The present study has some limitations especially in relation to the use of stents, which would be best addressed by a randomized controlled study. In addition, TDT used in this study is not available in the US or in the European countries, although it is sold commercially in Japan.

In conclusion, the TDT placement following urgent colonoscopy was effective in the management of acute left-sided colorectal obstruction. In the majority of patients the materials and methods used for the TDT placement were simple and easy.

## Abbreviations

SEMS: self-expanding metallic stents
TDT: transanal drainage tube
